# Disrupted White Matter Integrity and Structural Brain Networks in Temporal Lobe Epilepsy With and Without Interictal Psychosis

**DOI:** 10.3389/fneur.2020.556569

**Published:** 2020-09-24

**Authors:** Daichi Sone, Noriko Sato, Yoko Shigemoto, Yukio Kimura, Norihide Maikusa, Miho Ota, Jacqueline Foong, Matthias Koepp, Hiroshi Matsuda

**Affiliations:** ^1^Integrative Brain Imaging Center, National Center of Neurology and Psychiatry, Tokyo, Japan; ^2^Department of Clinical and Experimental Epilepsy, University College London Institute of Neurology, London, United Kingdom; ^3^Department of Radiology, National Center of Neurology and Psychiatry, Tokyo, Japan; ^4^Division of Clinical Medicine, Department of Neuropsychiatry, Faculty of Medicine, University of Tsukuba, Ibaraki, Japan

**Keywords:** interictal psychosis, temporal lobe epilepsy, graph theory, white matter microstructure, diffusion tensor imaging

## Abstract

**Background:** Despite the importance of psychosis as a comorbidity of temporal lobe epilepsy (TLE), the underlying neural mechanisms are still unclear. We aimed to investigate abnormalities specific to psychosis in TLE, using diffusion MRI parameters and graph-theoretical network analysis.

**Material and Methods:** We recruited 49 patients with TLE (20 with and 29 without interictal schizophrenia-like psychosis) and 42 age-/gender-matched healthy controls. We performed 3-tesla MRI scans including 3D T1-weighted imaging and diffusion tensor imaging in all participants. Among the three groups, fractional anisotropy (FA), mean diffusivity (MD), and global network metrics were compared by analyses of covariance. Regional connectivity strength was compared by network-based statistics.

**Results:** Compared to controls, TLE patients showed significant temporal and extra-temporal changes in FA, and MD, which were more severe and widespread in patients with than without psychosis. We observed distinct differences between TLE patients with and without psychosis in the anterior thalamic radiation (ATR), inferior fronto-occipital fasciculus (IFOF), and inferior longitudinal fasciculus (ILF). Similarly, for network metrics, global, and local efficiency and increased path length were significantly reduced in TLE patients compared to controls, but with more severe changes in TLE with psychosis than without psychosis. Network-based statistics detected significant differences between TLE with and without psychosis mainly involving the left limbic and prefrontal areas.

**Conclusion:** TLE patients with interictal schizophrenia-like psychosis showed more widespread and severe white matter impairment, involving the ATR, IFOF and ILF, as well as disrupted network connectivity, particularly in the left limbic and prefrontal cortex, than patients without psychosis.

## Introduction

Epilepsy is a common neurological disorder, which affects ~50 million people world-wide (1). While its defining criteria are epileptic seizures, the definition of epilepsy also encompasses psychiatric comorbidities and behavioral problems (2), which may have a greater impact on patients' quality of life than seizures (3). In patients with epilepsy, psychosis is 7.8 times more prevalent than in the general population (4). Despite this high prevalence and effect on quality of life, the pathophysiology of psychosis in epilepsy is largely unknown. Moreover, there is a bidirectional relationship between epilepsy and psychiatric comorbidities (2). In addition to the high incidence, psychiatric symptoms sometimes occur prior to epilepsy and predict poor seizure outcome following epilepsy surgery (5). Thus, to investigate the mechanisms of psychiatric comorbidities in epilepsy may also deepen our biological understanding of epilepsy.

In epilepsy, particularly in temporal lobe epilepsy (TLE), patients develop psychosis typically over 10 years after the onset of epilepsy (6). The prevalence in TLE is high, up to 19% (7). Several neuroimaging studies tried to clarify the pathological changes of psychosis in epilepsy (8). A few studies using structural MRI suggested hippocampal tail atrophy (9) as well as abnormal aging (10) in TLE patients with psychosis. However, according to a recent systematic review (8), the results of morphological studies are not consistent, and little is known about other advanced imaging modalities.

Diffusion tensor imaging (DTI) is used to evaluate white matter tracts through microstructural parameters such as fractional anisotropy (FA) and mean diffusivity (MD) (11). A previous DTI study on interictal psychosis in TLE reported decreased FA and increased MD within the limited region of interests (ROI) in frontal and temporal lobes (12). Another study reported abnormal brain network metrics by using the gray matter covariance method (13). However, there is still no study investigating whole-brain white matter integrity nor the brain structural networks based on DTI in psychosis in epilepsy, which may possibly yield specific abnormalities as well as potential biomarkers. In addition, recent technical advances enable us to examine brain networks and structural connectivity (14). Network analysis including graph-theory has been used in both epilepsy (15) and psychiatric disorders (14).

In this study, we used DTI and graph theoretical network analysis to investigate white matter integrity and neural networks in TLE with and without interictal psychosis.

## Materials and Methods

### Participants

Between November 2013 and December 2017, we recruited 20 consecutive patients with TLE and interictal schizophrenia-like psychosis, including auditory hallucination and delusion, (TLE-P group) at our institute. TLE diagnosis was based on the following criteria: (1) focal seizures consistent with TLE; and (2) focal epileptiform discharges predominantly in temporal areas in conventional scalp electroencephalography. The laterality of the focus was determined based on clinical symptoms, interictal epileptiform discharge, and visual assessment of MRI. Using the Diagnostic and Statistical Manual of Mental Disorders, 4th edition criteria (16), a board-certified psychiatrist diagnosed interictal psychosis by an interview. We also recruited, as patient controls, 29 age-, gender-, and laterality-matched TLE patients without any history of psychotic episodes (TLE-NonP group). The criteria for TLE diagnosis was same, and the lack of psychotic episodes was confirmed based on an interview with a board-certified psychiatrist. In addition, 42 age- and gender-matched healthy subjects were also enrolled as healthy controls (HC group), based on the following criteria: no history of neurological or psychiatric diseases; and no medication of central nervous system agents.

Subjects were excluded using the following criteria: (1) a history of acute encephalitis, meningitis, severe head trauma, or ischemic encephalopathy; (2) epileptogenic lesions, such as tumor or cortical dysplasia, except for unilateral hippocampal sclerosis; (3) epileptiform activity in extra-temporal regions on electroencephalography; (4) severe mood, personality, or developmental disorders; or (5) drug/substance-induced psychoses.

We collected the following clinical data for all patients: gender, age, onset age of seizure and psychosis, duration from seizure onset, types of seizures, frequency of focal impaired awareness seizures (FIAS) per month, and anti-epileptic drugs (AEDs). All participants gave written informed consent, and this study was approved by the Institutional Review Board at the National Center of Neurology and Psychiatry Hospital.

### MRI Acquisition

All MRI scans were performed on a 3.0-T MR system with a 32-channel coil (Philips Medical Systems, Best, The Netherlands). The parameters of the DTI sequence were as following: repetition time (TR), 6,700 ms; echo time (TE), 58 ms; flip angle, 90°; effective slice thickness, 3.0 mm with no gap; slices, 60; matrix, 80 × 78; and field of view (FOV), 24 × 24 cm. The DTI was acquired along 15 non-collinear directions with a diffusion-weighted factor b of 1,000 s/mm^2^, and one image was acquired without diffusion gradient. To increase the signal-to-noise ratio, we adopted the number of excitations (NEX) of 2 for DTI acquisition.

Three-dimensional (3D) sagittal T1-weighted images were obtained by the following protocol: TR, 7.12 ms; TE, 3.4 ms; flip angle, 10°; NEX, 1; effective slice thickness, 0.6 mm with no gap; slices, 300; matrix, 260 × 320; and FOV, 26 × 24 cm. Additionally, transverse turbo spin echo T2-weighted imaging and coronal fluid-attenuated inversion recovery (FLAIR) imaging were also obtained for visual MRI assessment.

### Tract-Based Spatial Statistics

Initially, we processed the DTI data with the PANDA toolbox v.1.3.1 (https://www.nitrc.org/projects/panda/) (17) running within the MATLAB 2018b and FMRIB Software Library (FSL) version 5.0.11. After eddy current correction and brain extraction, the software provided voxel-wise maps of FA and MD for each participant. To compare FA and MD maps among the three groups, we performed tract-based spatial statistics (TBSS) (11) and atlas-based ROI analysis using all the tracts of the Johns Hopkins University atlas (18). The PANDA toolbox calculated mean FA or MD values within each tract of the atlas in each patient.

### Network Analysis

The pipelines and settings for network analysis were the same as in our previous study (19).

From the DTI data, the PANDA toolbox constructed individuals' structural white matter networks using the number of fibers as deterministic, weighted edges. All the tracts were computed by seeding each voxel with an FA that was >0.2. We also used individuals' 3D-T1 images for definition of network nodes; we co-registered each FA image to its corresponding 3D-T1 image using affine transformation and then non-linearly transformed the 3D-T1 image to the MNI space. Thereafter, the standard atlases of gray mattes were warped to the native space of each participant and used for definition of nodes. For the network construction process, the details are shown in the paper of the PANDA toolbox (17).

We used two different atlases for parcellation schemes of graph theoretical analysis, in order to enhance validity and reproducibility: the Automated Anatomical Labeling (AAL) atlas with 90 regions of interest (ROIs) (20) and the Harvard-Oxford atlas (HOA) with 110 ROIs (21). Supplementary Tables 1, 2 show the ROIs' names and locations.

To perform graph theoretical analysis, we used the GRETNA toolbox (https://www.nitrc.org/projects/gretna/) (22). As global network properties, the GRETNA toolbox analyzed the small-worldness property (clustering coefficient and characteristic path length) and global network efficiency (global efficiency and local efficiency) of each subject's brain. The definitions and meanings of these network properties are introduced elsewhere (23). For local network evaluation, we used a network-based statistic (NBS) method to detect edges with significantly altered connectivity strengths. For network visualization, the BrainNet Viewer (http://www.nitrc.org/projects/bnv/) (24) was utilized.

### Statistics

For the comparison of the FA/MD values and the global network metrics among the three groups, analysis of covariance (ANCOVA) with age and gender as covariates and *post-hoc* comparisons based on the estimated marginal means with Bonferroni's correction were used with SPSS software version 25.0. Moreover, between TLE-P and TLE-NonP groups, we also performed additional ANCOVA comparisons with age, gender, number of AEDs, and frequency of FIAS as covariates. The SPSS software was also used to analyze the clinical parameters. For the local connectivity strengths, we used NBS correction for multiple comparison by the GRETNA toolbox. A two-sided *p* < 0.05 was deemed significant.

## Results

### Clinical Demographics

The demographics of the three groups are described in Table 1. There were no significant differences in age, gender, disease duration, and other clinical features. Two patients were seizure free with AEDs in TLE-P, while all patients were drug-resistant in TLE-NonP.

**Table 1:** Demographics of patients with TLE with and without interictal psychosis and healthy subjects.

**Feature**	**TLE-P (*n* = 20)**	**TLE-NonP (*n* = 29)**	**Healthy (*n* = 42)**	***p*-value**
**Gender (no.)**				
Men:women	9:11	15:14	21:21	0.89^*^
**Age at the examination (years)**				
Mean ± SD	46.4 ± 12.0	45.5 ± 11.7	45.8 ± 11.2	0.97^†^
**Disease duration (years)**				
Mean onset age ± SD	16.5 ± 14.8	16.2 ± 8.7	N/A	0.96^‡^
Mean duration of epilepsy ± SD	29.9 ± 13.4	29.3 ± 12.6	N/A	0.87^‡^
Mean onset age of psychosis ± SD	32.2 ± 16.2	N/A	N/A	N/A
Mean duration of psychosis ± SD	14.2 ± 12.8	N/A	N/A	N/A
**Clinical features**				
Focus side (L:R)	9:11	14:15	N/A	0.82^*^
Hippocampal sclerosis (no.)	10	15	N/A	0.91^*^
Patients with FBTCS (no.)	3	4	N/A	0.91^*^
Patients with FAS (no.)	8	13	N/A	0.92^*^
Mean frequency of FIAS per month (no.)	3.15 ± 6.61	4.35 ± 6.00	N/A	0.51^‡^
Mean number of AEDs ± SD	2.25 ± 1.02	2.66 ± 1.01	N/A	0.18^‡^

*TLE-P, TLE with psychosis; TLE-NonP, TLE without psychosis group; FBTCS, focal to bilateral tonic-clonic seizures; FAS, focal aware seizures; FIAS, focal impaired awareness seizures; AEDs, anti-epileptic drugs; N/A, not available*.

* *χ^2^ test*,

† *one-way ANOVA*,

‡ *unpaired t-test*.

### DTI Metrics

**Figure 1:**
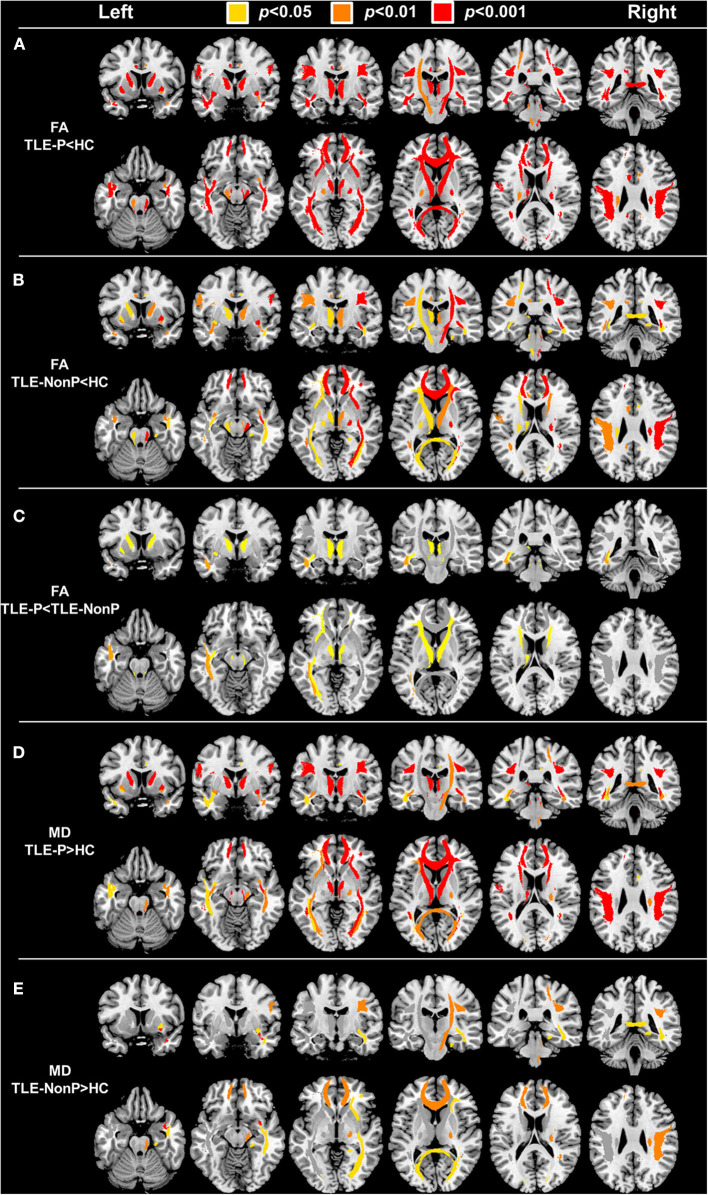
The results of atlas-based TBSS comparison among the TLE-P, TLE-NonP, and HC groups. Colored tracts denote significant group differences. **(A)** TLE-P < HC in FA, **(B)** TLE-NonP < HC in FA, **(C)** TLE-NonP < TLE-P in FA, **(D)** TLE-P>HC in MD, and **(E)** TLE-NonP>HC in MD.

The results of group comparison in FA and MD are shown in Table 2 and Figure 1. Whereas, TLE-NonP showed reduced FA and increased MD in temporal and extra-temporal areas, more widespread and severe abnormalities were found in TLE-P. Directly comparing TLE-P and TLE-NonP groups, we found significant differences in anterior thalamic radiation (ATR), inferior fronto-occipital fasciculus (IFOF), and inferior longitudinal fasciculus (ILF). Furthermore, with additional covariates of AEDs and seizure frequency, the differences remained significant (Table 3).

**Table 2:** The results of FA and MD based on TBSS analysis among the three groups.

	**FA**		**MD** **×** **10**^****3****^					
	**TLE-P**	**TLE-NonP**	**HC**	**F-val**.	**TLE-P**	**TLE-NonP**	**HC**	***F*-value**
ATR-left	**0.29** **±** 0.027^***^^^†^^	**0.31** **±** 0.024^*^	0.32 ± 0.013	15.82	**1.02** **±** 0.191^***^	0.95 ± 0.142	0.89 ± 0.075	9.56
ATR-right	**0.28** **±** 0.026^***^^^†^^	**0.29** **±** 0.027^**^	0.31 ± 0.015	20.20	**1.01** **±** 0.163^***^	0.96 ± 0.150	0.89 ± 0.078	10.19
CST-left	**0.45** **±** 0.026^**^	**0.46** **±** 0.027^*^	0.47 ± 0.020	7.95	0.81 ± 0.045	0.80 ± 0.037	0.79 ± 0.029	1.69
CST-right	**0.45** **±** 0.023^***^	**0.46** **±** 0.024^***^	0.48 ± 0.019	14.18	**0.82** **±** 0.037^**^	**0.82** **±** 0.037^**^	0.80 ± 0.024	7.43
Cing(C)-left	**0.36** **±** 0.034^***^	**0.38** **±** 0.025^**^	0.40 ± 0.021	15.39	0.84 ± 0.049	0.83 ± 0.051	0.82 ± 0.040	1.91
Cing(C)-right	**0.32** **±** 0.034^**^	**0.33** **±** 0.031^*^	0.34 ± 0.022	8.39	**0.81** **±** 0.046^*^	0.79 ± 0.048	0.78 ± 0.041	3.62
Cing(H)-left	0.24 ± 0.034	0.24 ± 0.029	0.25 ± 0.027	3.12	0.85 ± 0.077	0.85 ± 0.080	0.82 ± 0.049	3.43
Cing(H)-right	0.23 ± 0.028	**0.23** **±** 0.034^*^	0.24 ± 0.026	3.60	0.89 ± 0.090	**0.91** **±** 0.117^*^	0.86 ± 0.045	4.56
Forceps major	**0.43** **±** 0.034^***^	**0.44** **±** 0.035^*^	0.46 ± 0.020	9.15	**0.92** **±** 0.080^**^	**0.90** **±** 0.080^*^	0.85 ± 0.049	7.64
Forceps minor	**0.32** **±** 0.024^***^	**0.33** **±** 0.024^***^	0.35 ± 0.016	21.60	**0.95** **±** 0.061^***^	**0.93** **±** 0.057^**^	0.89 ± 0.042	13.58
IFOF-left	**0.32** **±** 0.023^***^^^†^^	**0.33** **±** 0.022^*^	0.35 ± 0.015	17.30	**0.85** **±** 0.061^**^	0.83 ± 0.039	0.82 ± 0.030	6.29
IFOF-right	**0.32** **±** 0.027^***^	**0.33** **±** 0.022^***^	0.35 ± 0.016	16.16	**0.86** **±** 0.055^***^	**0.84** **±** 0.043^*^	0.82 ± 0.028	10.80
ILF-left	**0.32** **±** 0.024^***^^††^	0.34 ± 0.021	0.35 ± 0.016	14.41	**0.84** **±** 0.062^*^	0.81 ± 0.027	0.80 ± 0.028	4.49
ILF-right	**0.35** **±** 0.023^***^	**0.36** **±** 0.023^*^	0.37 ± 0.019	10.99	**0.83** **±** 0.041^**^	**0.82** **±** 0.042^*^	0.80 ± 0.024	7.19
SLF-left	**0.30** **±** 0.021^***^	**0.31** **±** 0.019^**^	0.32 ± 0.012	15.57	**0.82** **±** 0.047^***^	0.79 ± 0.040	0.78 ± 0.031	9.46
SLF-right	**0.30** **±** 0.021^***^	**0.31** **±** 0.021^***^	0.33 ± 0.017	14.16	**0.81** **±** 0.046^***^	**0.80** **±** 0.041^**^	0.77 ± 0.026	12.70
UF-left	**0.29** **±** 0.031^***^	**0.30** **±** 0.023^**^	0.32 ± 0.018	12.21	**0.90** **±** 0.092^*^	0.87 ± 0.045	0.86 ± 0.034	3.84
UF-right	**0.28** **±** 0.027^**^	**0.28** **±** 0.027^**^	0.30 ± 0.020	10.40	**0.91** **±** 0.057^**^	**0.92** **±** 0.071^***^	0.87 ± 0.034	10.27
SLF(T)-left	**0.37** **±** 0.041^***^	**0.39** **±** 0.030^***^	0.40 ± 0.024	8.03	**0.80** **±** 0.048^***^	0.77 ± 0.032	0.76 ± 0.027	8.25
SLF(T)-right	**0.42** **±** 0.035^**^	0.44 ± 0.035	0.45 ± 0.030	6.60	**0.80** **±** 0.047^*^	0.79 ± 0.045	0.77 ± 0.027	3.51

*ATR, anterior thalamic radiation; CST, corticospinal tract; Cing(C), cingulum (cingulate gyrus); Cing(H), cingulum (hippocampus); IFOF, inferior fronto-occipital fasciculus; ILF, inferior longitudinal fasciculus; SLF, superior longitudinal fasciculus; UF, uncinate fasciculus; SLF(T), superior longitudinal fasciculus (temporal part)*.

*Bold font denotes significant changes compared with HC*.

* *p < 0.05*,

** *p < 0.01*,

*** *p < 0.001, compared with HC*.

† *p < 0.05*,

†† *p < 0.01, between TLE-P and TLE-NonP*.

**Table 3:** Results from additional ANCOVA comparisons in FA and MD between TLE-P and TLE-NonP groups with age, gender, number of AEDs, and frequency of FIAS as covariates.

	**FA**				**MD** **×** **10**^****3****^			
	**Estimated marginal means in TLE-P**	**Estimated marginal means in TLE-NonP**	***F*-value**	***p*-value**	**Estimated marginal means in TLE-P**	**Estimated marginal means in TLE-NonP**	***F*-value**	***p*-value**
ATR-left	**0.29**	**0.31**	**5.43**	**0.024**	1.02	0.95	2.83	0.100
ATR-right	**0.28**	**0.30**	**6.97**	**0.011**	1.01	0.95	2.53	0.119
CST-left	0.45	0.46	3.36	0.074	0.81	0.80	1.28	0.265
CST-right	0.45	0.46	2.82	0.101	0.82	0.82	0.27	0.603
Cing(C)-left	0.36	0.38	3.46	0.070	0.85	0.83	1.94	0.171
Cing(C)-right	0.32	0.33	1.27	0.265	0.81	0.79	2.13	0.152
Cing(H)-left	0.24	0.24	0.00	0.949	0.85	0.85	0.03	0.859
Cing(H)-right	0.23	0.23	0.01	0.922	0.90	0.91	0.20	0.657
Forceps major	0.43	0.45	3.04	0.088	0.92	0.90	1.11	0.298
Forceps minor	**0.32**	**0.33**	**5.65**	**0.022**	0.96	0.93	3.63	0.063
IFOF-left	**0.32**	**0.34**	**6.41**	**0.015**	0.85	0.83	3.21	0.080
IFOF-right	0.32	0.33	3.38	0.073	0.86	0.84	2.37	0.131
ILF-left	**0.32**	**0.34**	**8.37**	**0.006**	0.84	0.81	3.53	0.067
ILF-right	**0.34**	**0.36**	**5.22**	**0.027**	0.83	0.82	0.63	0.430
SLF-left	0.30	0.31	2.78	0.102	**0.82**	**0.79**	**4.50**	**0.040**
SLF-right	0.30	0.31	2.39	0.129	0.82	0.80	2.52	0.120
UF-left	0.29	0.30	1.81	0.186	0.91	0.87	2.90	0.096
UF-right	0.28	0.28	0.43	0.514	0.91	0.92	0.08	0.777
SLF(T)-left	**0.37**	**0.39**	**5.61**	**0.022**	0.80	0.77	3.12	0.085
SLF(T)-right	0.42	0.44	3.80	0.058	0.80	0.79	0.57	0.453

*ATR, anterior thalamic radiation; CST, corticospinal tract; Cing(C), cingulum (cingulate gyrus); Cing(H), cingulum (hippocampus); IFOF, inferior fronto-occipital fasciculus; ILF, inferior longitudinal fasciculus; SLF, superior longitudinal fasciculus; UF, uncinate fasciculus; SLF(T), superior longitudinal fasciculus (temporal part)*.

*Bold font denotes significant changes between TLE-P and TLE-NonP*.

### Global Network Metrics

**Figure 2:**
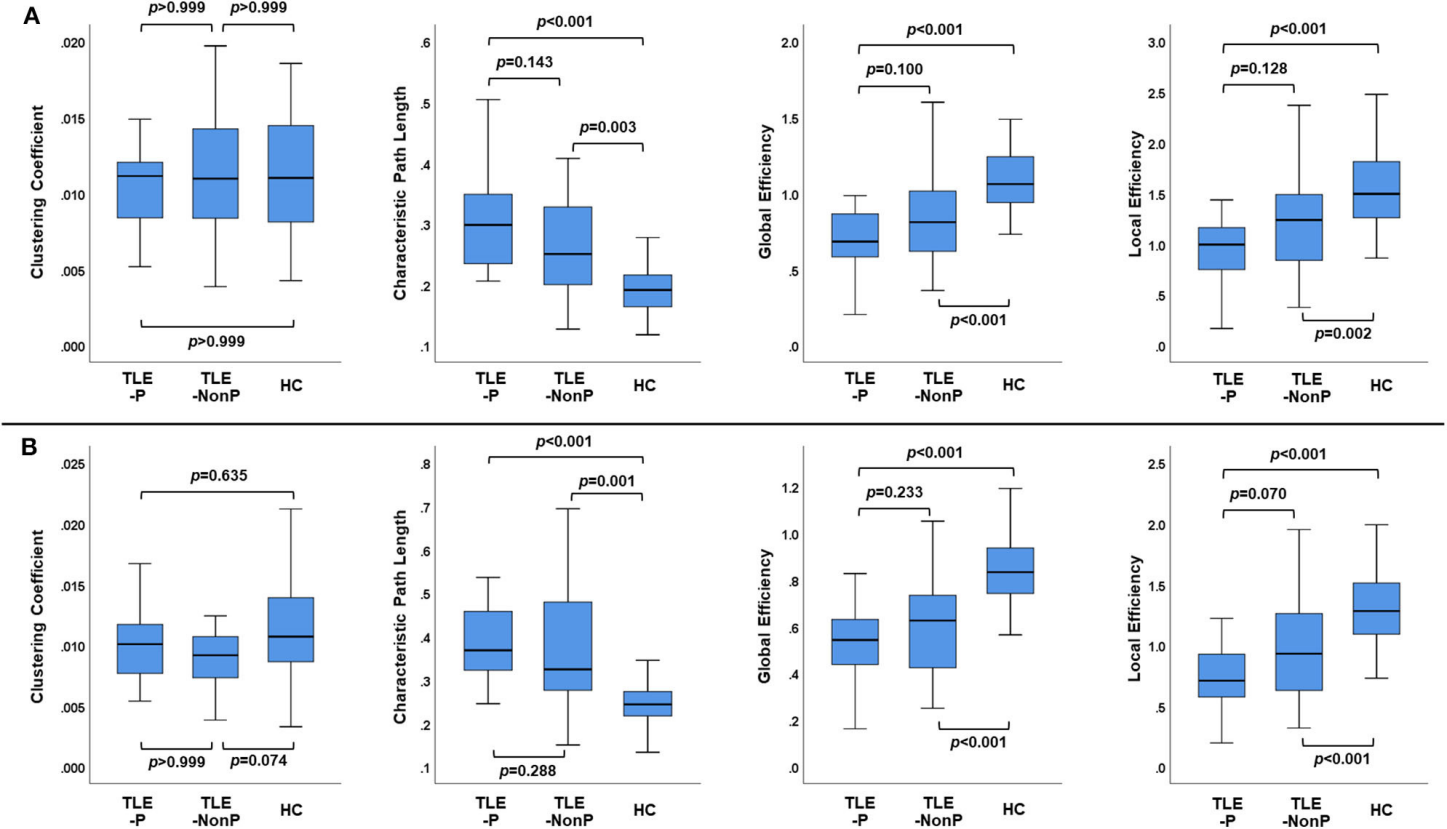
The results of global network metrics among the TLE-P, TLE-NonP, and HC groups based on AAL **(A)** and HOA **(B)** atlases.

Figure 2 presents the results of global network metrics comparison. Whilst there is no significant difference in clustering coefficient, both TLE groups showed significantly increased path length and decreased global and local network efficiency, compared with HC. This result was reproduced by the two different schemes. Although network efficiency in TLE-P tended to be lower than TLE-NonP, the difference was not significant. After correction for AEDs and seizure frequency, we observed significantly reduced network efficiency and increased path length in TLE-P compared to TLE-NonP (Table 4).

**Table 4:** Results from additional ANCOVA comparisons in global network metrics between TLE-P and TLE-NonP groups with age, gender, number of AEDs, and frequency of FIAS as covariates.

	**Estimated marginal means in TLE-P**	**Estimated marginal means in TLE-NonP**	***F*-value**	***p*-value**
**AAL parcellation**				
Clustering coefficient	0.010	0.011	0.59	0.446
Characteristic path length	**0.349**	**0.265**	**6.30**	**0.016**
Global efficiency	**0.661**	**0.865**	**8.16**	**0.007**
Local efficiency	**0.890**	**1.240**	**10.14**	**0.003**
**HOA parcellation**				
Clustering coefficient	0.010	0.009	0.52	0.475
Characteristic path length	**0.460**	**0.357**	**4.95**	**0.031**
Global efficiency	**0.508**	**0.659**	**6.05**	**0.018**
Local efficiency	**0.700**	**1.006**	**9.06**	**0.004**

*Bold font denotes significant changes between TLE-P and TLE-NonP*.

### Local Connectivity

**Figure 3:**
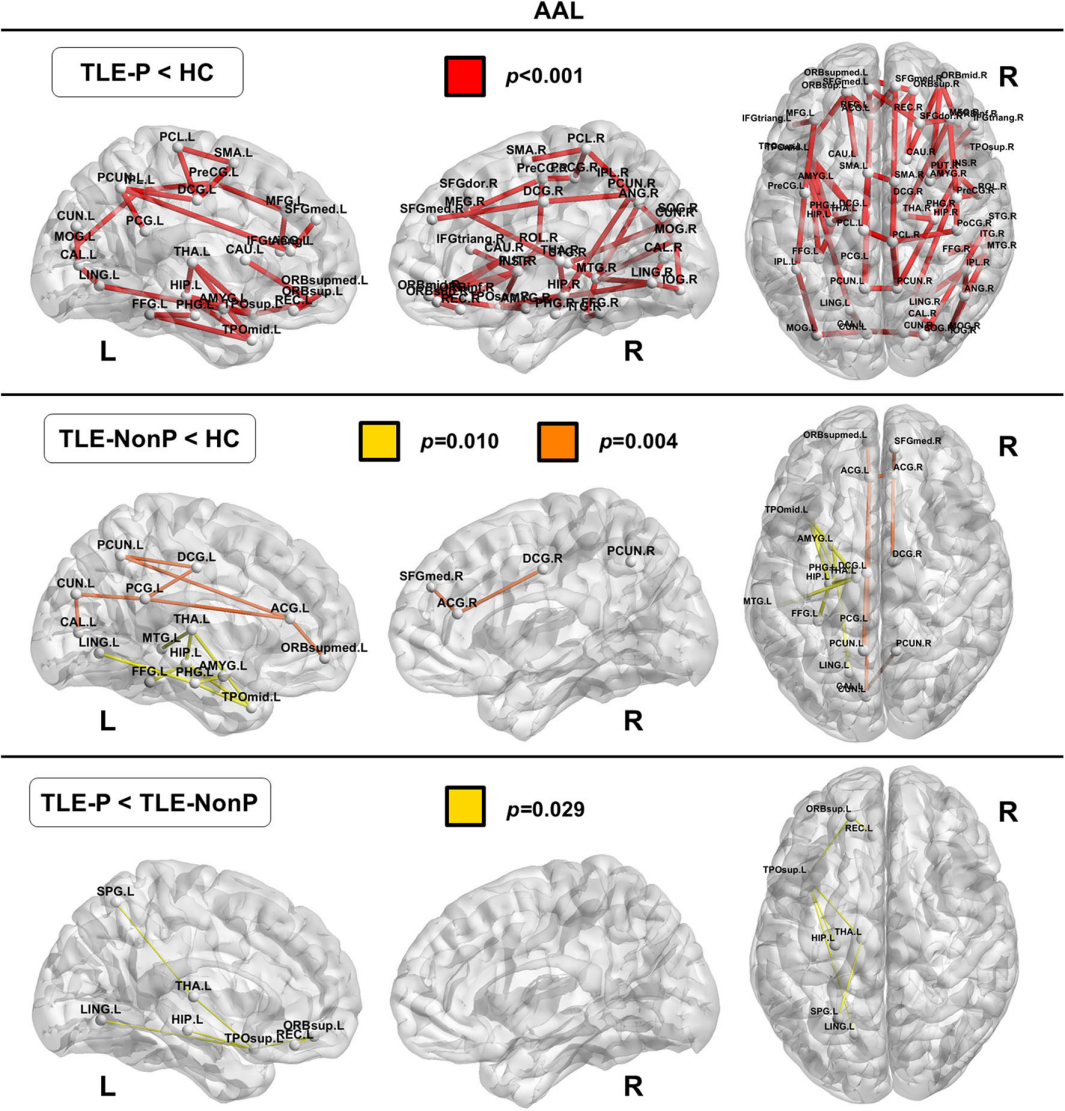
The results of connectivity strength analysis based on network-based statistics in the AAL parcellation. Colored network connectivity denotes significant differences. The node abbreviations correspond to those of Supplementary Table 1.

**Figure 4:**
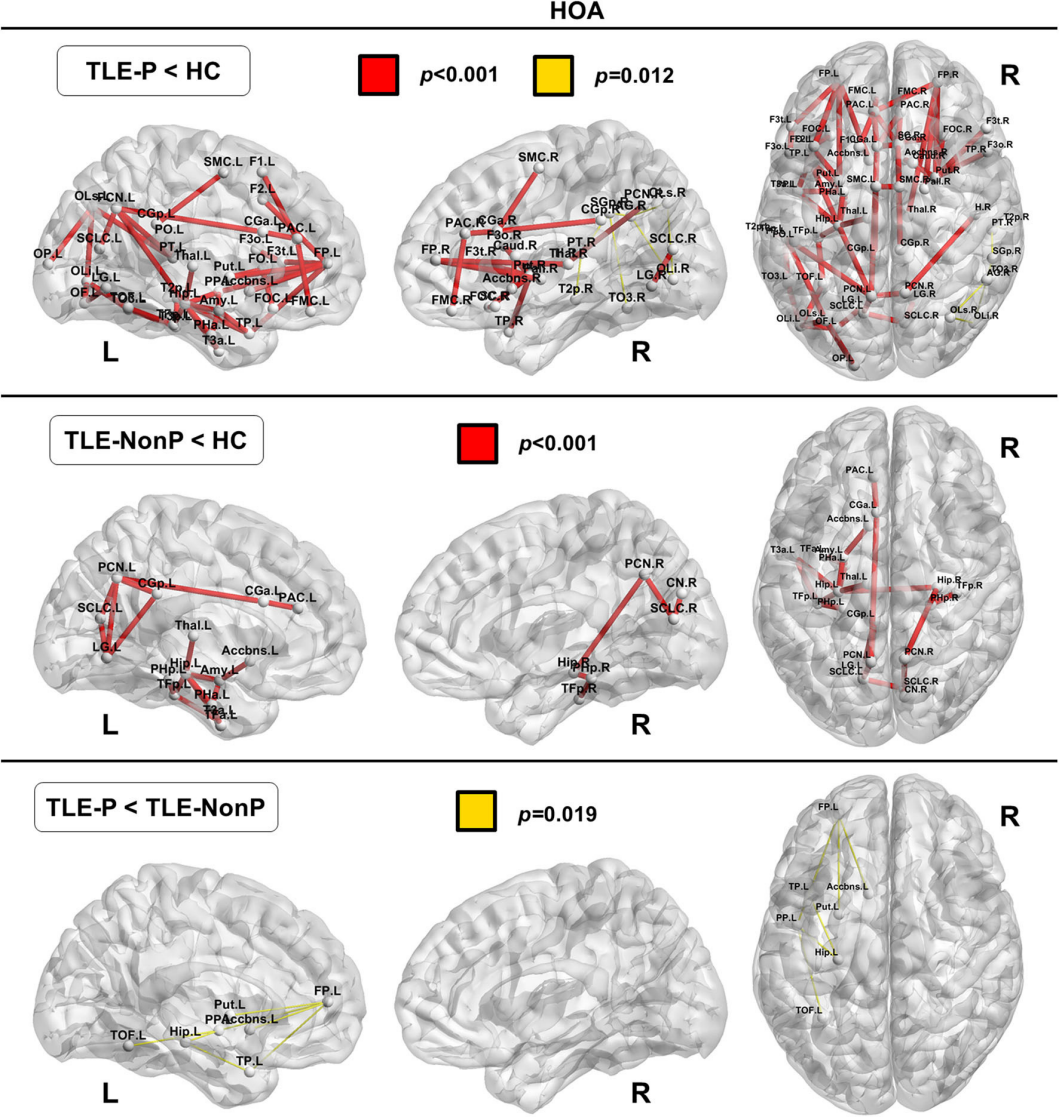
The results of connectivity strength analysis based on network-based statistics in the HOA parcellation. Colored network connectivity denotes significant differences. The node abbreviations correspond to those of Supplementary Table 2.

The regional network strength was analyzed with NBS (Figures 3, 4). The list of involved nodes can be found in Supplementary Table 3. Both TLE groups showed reduced network connectivity compared to HC, but the network of the TLE-P group was more widely and severely impaired than the TLE-nonP group. Additionally, the direct comparison between the two TLE groups revealed significant network differences mainly involving the left limbic and prefrontal areas. These results were consistent across the two different parcellation schemes.

## Discussion

Using graph-theoretical brain structural networks as well as TBSS analysis of DTI data, we found disrupted white matter integrity and network impairment in patients with TLE, with more disrupted white matter integrity and network connectivity in TLE with psychosis than without. The effect of interictal psychosis was particularly pronounced in ATR, IFOF, and ILF, with regional connectivity being markedly impaired in the left limbic and prefrontal areas. These graph-theoretical findings were reproduced using two different schemes. To our knowledge, this is the first study examining whole-brain DTI and white matter structural networks in psychosis in epilepsy.

Previously, we reported reduced FA in the bilateral temporal and frontal lobes and increased MD in the bilateral temporal lobe in interictal psychosis in TLE (12). Whereas, our previous findings were based on manually delineated four ROIs, we now extended our study to include whole-brain and tract-based analysis and healthy data. Specifically, the ATR, IFOF, and ILF may be relevant for the pathophysiology of psychotic symptoms in TLE: the ATR is the main projection connecting the prefrontal cortex and the thalamus through the anterior limb of the internal capsule (25), whereas the IFOF and ILF are connecting the temporal lobe, parieto-occipital area, and frontal lobe (26, 27). Thus, the ATR is involved in the thalamo-frontal neural networks, and the IFOF and ILF are both associated with limbic structures (28). Considering the roles of these structures and their relevance for emotion, memory, or behavior, these areas are also likely to be relevant for the pathophysiology of psychosis.

In schizophrenia, the most frequently reported abnormal white matter tracts are the uncinate fasciculus, cingulum, corpus callosum, and corticospinal tract (29). These fibers are also damaged in TLE without psychosis (Table 2 and Figure 1). Thus, psychosis in epilepsy may have a different pathophysiology from schizophrenia, which might explain the different prevalence of psychosis in epilepsy and the general population. Further investigation using additional comparison with schizophrenia patients would expand our knowledge on this point.

Significantly longer path length and reduced network efficiency have been reported consistently in schizophrenia when compared to healthy subjects (30–32). In a previous study using gray matter covariance network analysis we reported decreased network efficiency in TLE patients with psychosis (13). Although the current study showed the same tendency, the difference between TLE with and without psychosis was not as marked as between schizophrenia and healthy controls. Both TLE groups showed significantly increased path length and decreased efficiency compared to HC, which is consistent with previous findings in TLE across different modalities (33). Whilst such inefficient brain networks may represent characteristic neural abnormalities in TLE, these network metrics are not specific, neither for TLE nor psychosis, given the subtle differences between TLE with and without psychosis.

Recent neuroimaging advances revealed abnormal aging processes of the brain in various neuropsychiatric disorders (34). Whereas, conventional gray matter morphometry yielded inconsistent results in psychosis of epilepsy (8), our previous study revealed an increased brain age of 10.9 years in TLE patients with psychosis, which was significantly higher than in TLE patients without psychosis (5.3 years) (10). According to a previous study on structural connectome and normal aging (35), both global and local efficiency of the brain tend to gradually decrease along with aging after approximately the third decade. Considering the age range of our cohort (i.e., late 40's on average), the tendency of network inefficiency in psychosis, despite its insignificance, might be related to the pathological aging process.

We also detected regional connectivity differences between the three groups using NBS analysis. The impairment was more widespread and severe in TLE patients with psychosis than without psychosis with differences found mainly in the left limbic and prefrontal areas (Figure 3), which is consistent with the abnormalities detected in the left IFOF and ILF. In fact, these areas and tracts are most often disrupted in schizophrenia (30, 32, 36), although schizophrenia shows even more widely impaired connectivity compared to healthy subjects.

Our study has several limitations. Firstly, the sample size was relatively small compared with other psychiatric cohort studies. However, in this small but well-defined group we detected several statistically significant and concordant findings. Secondly, consistent with other neuroimaging studies on psychosis in epilepsy, we analyzed both left and right TLE patients together (12, 37–39), focusing on whether psychosis was ever present or nor. However, the side of the focus might also affect white matter findings in TLE (40). The proportion of laterality was nearly the same in our two TLE cohorts. Future investigations would require larger sample sizes and subdivide analysis according to laterality of focus. Thirdly, given the relatively low number of DTI directions (i.e., 15), which may have caused poorer evaluation in crossing fibers reflected in low FA, more accurate MRI protocols can now be employed. Furthermore, medication effects need to be factored in when comparing our two cohorts. Whilst we controlled for the effects of anti-epileptic drugs, we cannot rule out that the observed changes in this cross-sectional analysis are not due to effects of anti-psychotic medication. Longitudinal studies are required to disentangle these effects. Additionally, in general, symptoms of psychosis are diverse, including delusion, auditory hallucination, disorganized speech, or catatonic behavior. It is also important to differentiate psychiatric symptoms from psychic aura caused by epileptic seizures in TLE. Thus, the lack of more detailed psychiatric evaluation might have led to confounding results, although our findings were generally compatible with past literature. Finally, the accumulated alpha errors due to multiple comparisons should be kept in mind.

## Conclusions

TLE patients with interictal psychosis showed more widespread and severe white matter impairment as well as disrupted network connectivity than TLE patients without psychosis. Specifically, the ATR, IFOF and ILF as well as the left limbic and prefrontal connectivity may have significant roles in the pathophysiology of psychosis in epilepsy. These findings may contribute to a better understanding and future development of imaging biomarker of psychosis in epilepsy.

## Data Availability Statement

The raw data supporting the conclusions of this article will be made available by the authors, without undue reservation.

## Ethics Statement

The studies involving human participants were reviewed and approved by the Institutional Review Board at the National Center of Neurology and Psychiatry Hospital. The patients/participants provided their written informed consent to participate in this study.

## Author Contributions

DS organized the whole study. DS, NS, and HM were involved in the study concept and design. DS, NS, YS, and YK performed recruitment and data acquisition. NM and DS analyzed the data. DS wrote the manuscript. NS, MO, JF, MK, and HM contributed to critical supervision. All authors read and approved the submitted version.

## Conflict of Interest

The authors declare that the research was conducted in the absence of any commercial or financial relationships that could be construed as a potential conflict of interest.
