# Supplementary material for: Disrupted White Matter Integrity and Structural Brain Networks in Temporal Lobe Epilepsy With and Without Interictal Psychosis
Source: Front Neurol. 2020 Sep 24;11:556569. doi: 10.3389/fneur.2020.556569 (PMC7542674; doi:10.3389/fneur.2020.556569)
Supplement: Supplementary file 1 [file Table_1.DOCX]

Supplementary Table 1. Regions of interest (ROIs) in the Automated Anatomical Labeling (AAL) Atlas.

| **Name of ROI** | **Abbreviation** | **Coordinates** |  |  |
| --- | --- | --- | --- | --- |
| 1 Precentral_L | PreCG.L | -38.65 | -5.68 | 50.94 |
| 2 Precentral_R | PreCG.R | 41.37 | -8.21 | 52.09 |
| 3 Frontal_Sup_L | SFGdor.L | -18.45 | 34.81 | 42.2 |
| 4 Frontal_Sup_R | SFGdor.R | 21.9 | 31.12 | 43.82 |
| 5 Frontal_Sup_Orb_L | ORBsup.L | -16.56 | 47.32 | -13.31 |
| 6 Frontal_Sup_Orb_R | ORBsup.R | 18.49 | 48.1 | -14.02 |
| 7 Frontal_Mid_L | MFG.L | -33.43 | 32.73 | 35.46 |
| 8 Frontal_Mid_R | MFG.R | 37.59 | 33.06 | 34.04 |
| 9 Frontal_Mid_Orb_L | ORBmid.L | -30.65 | 50.43 | -9.62 |
| 10 Frontal_Mid_Orb_R | ORBmid.R | 33.18 | 52.59 | -10.73 |
| 11 Frontal_Inf_Oper_L | IFGoperc.L | -48.43 | 12.73 | 19.02 |
| 12 Frontal_Inf_Oper_R | IFGoperc.R | 50.2 | 14.98 | 21.41 |
| 13 Frontal_Inf_Tri_L | IFGtriang.L | -45.58 | 29.91 | 13.99 |
| 14 Frontal_Inf_Tri_R | IFGtriang.R | 50.33 | 30.16 | 14.17 |
| 15 Frontal_Inf_Orb_L | ORBinf.L | -35.98 | 30.71 | -12.11 |
| 16 Frontal_Inf_Orb_R | ORBinf.R | 41.22 | 32.23 | -11.91 |
| 17 Rolandic_Oper_L | ROL.L | -47.16 | -8.48 | 13.95 |
| 18 Rolandic_Oper_R | ROL.R | 52.65 | -6.25 | 14.63 |
| 19 Supp_Motor_Area_L | SMA.L | -5.32 | 4.85 | 61.38 |
| 20 Supp_Motor_Area_R | SMA.R | 8.62 | 0.17 | 61.85 |
| 21 Olfactory_L | OLF.L | -8.06 | 15.05 | -11.46 |
| 22 Olfactory_R | OLF.R | 10.43 | 15.91 | -11.26 |
| 23 Frontal_Sup_Medial_L | SFGmed.L | -4.8 | 49.17 | 30.89 |
| 24 Frontal_Sup_Medial_R | SFGmed.R | 9.1 | 50.84 | 30.22 |
| 25 Frontal_Med_Orb_L | ORBsupmed.L | -5.17 | 54.06 | -7.4 |
| 26 Frontal_Med_Orb_R | ORBsupmed.R | 8.16 | 51.67 | -7.13 |
| 27 Rectus_L | REC.L | -5.08 | 37.07 | -18.14 |
| 28 Rectus_R | REC.R | 8.35 | 35.64 | -18.04 |
| 29 Insula_L | INS.L | -35.13 | 6.65 | 3.44 |
| 30 Insula_R | INS.R | 39.02 | 6.25 | 2.08 |
| 31 Cingulum_Ant_L | ACG.L | -4.04 | 35.4 | 13.95 |
| 32 Cingulum_Ant_R | ACG.R | 8.46 | 37.01 | 15.84 |
| 33 Cingulum_Mid_L | DCG.L | -5.48 | -14.92 | 41.57 |
| 34 Cingulum_Mid_R | DCG.R | 8.02 | -8.83 | 39.79 |
| 35 Cingulum_Post_L | PCG.L | -4.85 | -42.92 | 24.67 |
| 36 Cingulum_Post_R | PCG.R | 7.44 | -41.81 | 21.87 |
| 37 Hippocampus_L | HIP.L | -25.03 | -20.74 | -10.13 |
| 38 Hippocampus_R | HIP.R | 29.23 | -19.78 | -10.33 |
| 39 ParaHippocampal_L | PHG.L | -21.17 | -15.95 | -20.7 |
| 40 ParaHippocampal_R | PHG.R | 25.38 | -15.15 | -20.47 |
| 41 Amygdala_L | AMYG.L | -23.27 | -0.67 | -17.14 |
| 42 Amygdala_R | AMYG.R | 27.32 | 0.64 | -17.5 |
| 43 Calcarine_L | CAL.L | -7.14 | -78.67 | 6.44 |
| 44 Calcarine_R | CAL.R | 15.99 | -73.15 | 9.4 |
| 45 Cuneus_L | CUN.L | -5.93 | -80.13 | 27.22 |
| 46 Cuneus_R | CUN.R | 13.51 | -79.36 | 28.23 |
| 47 Lingual_L | LING.L | -14.62 | -67.56 | -4.63 |
| 48 Lingual_R | LING.R | 16.29 | -66.93 | -3.87 |
| 49 Occipital_Sup_L | SOG.L | -16.54 | -84.26 | 28.17 |
| 50 Occipital_Sup_R | SOG.R | 24.29 | -80.85 | 30.59 |
| 51 Occipital_Mid_L | MOG.L | -32.39 | -80.73 | 16.11 |
| 52 Occipital_Mid_R | MOG.R | 37.39 | -79.7 | 19.42 |
| 53 Occipital_Inf_L | IOG.L | -36.36 | -78.29 | -7.84 |
| 54 Occipital_Inf_R | IOG.R | 38.16 | -81.99 | -7.61 |
| 55 Fusiform_L | FFG.L | -31.16 | -40.3 | -20.23 |
| 56 Fusiform_R | FFG.R | 33.97 | -39.1 | -20.18 |
| 57 Postcentral_L | PoCG.L | -42.46 | -22.63 | 48.92 |
| 58 Postcentral_R | PoCG.R | 41.43 | -25.49 | 52.55 |
| 59 Parietal_Sup_L | SPG.L | -23.45 | -59.56 | 58.96 |
| 60 Parietal_Sup_R | SPG.R | 26.11 | -59.18 | 62.06 |
| 61 Parietal_Inf_L | IPL.L | -42.8 | -45.82 | 46.74 |
| 62 Parietal_Inf_R | IPL.R | 46.46 | -46.29 | 49.54 |
| 63 SupraMarginal_L | SMG.L | -55.79 | -33.64 | 30.45 |
| 64 SupraMarginal_R | SMG.R | 57.61 | -31.5 | 34.48 |
| 65 Angular_L | ANG.L | -44.14 | -60.82 | 35.59 |
| 66 Angular_R | ANG.R | 45.51 | -59.98 | 38.63 |
| 67 Precuneus_L | PCUN.L | -7.24 | -56.07 | 48.01 |
| 68 Precuneus_R | PCUN.R | 9.98 | -56.05 | 43.77 |
| 69 Paracentral_Lobule_L | PCL.L | -7.63 | -25.36 | 70.07 |
| 70 Paracentral_Lobule_R | PCL.R | 7.48 | -31.59 | 68.09 |
| 71 Caudate_L | CAU.L | -11.46 | 11 | 9.24 |
| 72 Caudate_R | CAU.R | 14.84 | 12.07 | 9.42 |
| 73 Putamen_L | PUT.L | -23.91 | 3.86 | 2.4 |
| 74 Putamen_R | PUT.R | 27.78 | 4.91 | 2.46 |
| 75 Pallidum_L | PAL.L | -17.75 | -0.03 | 0.21 |
| 76 Pallidum_R | PAL.R | 21.2 | 0.18 | 0.23 |
| 77 Thalamus_L | THA.L | -10.85 | -17.56 | 7.98 |
| 78 Thalamus_R | THA.R | 13 | -17.55 | 8.09 |
| 79 Heschl_L | HES.L | -41.99 | -18.88 | 9.98 |
| 80 Heschl_R | HES.R | 45.86 | -17.15 | 10.41 |
| 81 Temporal_Sup_L | STG.L | -53.16 | -20.68 | 7.13 |
| 82 Temporal_Sup_R | STG.R | 58.15 | -21.78 | 6.8 |
| 83 Temporal_Pole_Sup_L | TPOsup.L | -39.88 | 15.14 | -20.18 |
| 84 Temporal_Pole_Sup_R | TPOsup.R | 48.25 | 14.75 | -16.86 |
| 85 Temporal_Mid_L | MTG.L | -55.52 | -33.8 | -2.2 |
| 86 Temporal_Mid_R | MTG.R | 57.47 | -37.23 | -1.47 |
| 87 Temporal_Pole_Mid_L | TPOmid.L | -36.32 | 14.59 | -34.08 |
| 88 Temporal_Pole_Mid_R | TPOmid.R | 44.22 | 14.55 | -32.23 |
| 89 Temporal_Inf_L | ITG.L | -49.77 | -28.05 | -23.17 |
| 90 Temporal_Inf_R | ITG.R | 53.69 | -31.07 | -22.32 |
