# Supplementary material for: Disrupted White Matter Integrity and Structural Brain Networks in Temporal Lobe Epilepsy With and Without Interictal Psychosis
Source: Front Neurol. 2020 Sep 24;11:556569. doi: 10.3389/fneur.2020.556569 (PMC7542674; doi:10.3389/fneur.2020.556569)
Supplement: Supplementary file 2 [file Table_2.DOCX]

Supplementary Table 2. Regions of interest (ROIs) in the Harvard-Oxford Atlas (HOA).

| Name of ROI | Abbreviation | Coordinates |  |  |
| --- | --- | --- | --- | --- |
| 1 Frontal Pole_L | FP.L | -25.01 | 52.77 | 7.75 |
| 2 Frontal Pole_R | FP.R | 26.44 | 52 | 8.6 |
| 3 Insular Cortex_L | INS.L | -36.42 | 1.01 | 0.16 |
| 4 Insular Cortex_R | INS.R | 37.5 | 2.65 | -0.17 |
| 5 Superior Frontal Gyrus_L | F1.L | -14.56 | 17.96 | 56.57 |
| 6 Superior Frontal Gyrus_R | F1.R | 15.09 | 17.55 | 57.52 |
| 7 Middle Frontal Gyrus_L | F2.L | -38.15 | 18.31 | 42.01 |
| 8 Middle Frontal Gyrus_R | F2.R | 39.19 | 18.3 | 43.02 |
| 9 Inferior Frontal Gyrus, pars triangularis_L | F3t.L | -49.76 | 28.6 | 8.59 |
| 10 Inferior Frontal Gyrus, pars triangularis_R | F3t.R | 51.84 | 27.82 | 7.72 |
| 11 Inferior Frontal Gyrus, pars opercularis_L | F3o.L | -50.69 | 14.63 | 15.22 |
| 12 Inferior Frontal Gyrus, pars opercularis_R | F3o.R | 52.49 | 15.48 | 16.37 |
| 13 Precentral Gyrus_L | PRG.L | -34.28 | -11.71 | 49.18 |
| 14 Precentral Gyrus_R | PRG.R | 35.08 | -10.59 | 49.79 |
| 15 Temporal Pole_L | TP.L | -40.44 | 11.07 | -29.78 |
| 16 Temporal Pole_R | TP.R | 40.99 | 12.93 | -29.31 |
| 17 Superior Temporal Gyrus, anterior division_L | T1a.L | -56 | -3.79 | -8.14 |
| 18 Superior Temporal Gyrus, anterior division_R | T1a.R | 57.22 | -0.98 | -10.41 |
| 19 Superior Temporal Gyrus, posterior division_L | T1p.L | -62.37 | -29.14 | 3.86 |
| 20 Superior Temporal Gyrus, posterior division_R | T1p.R | 61.35 | -23.87 | 1.5 |
| 21 Middle Temporal Gyrus, anterior division_L | T2a.L | -57.8 | -4.41 | -22.05 |
| 22 Middle Temporal Gyrus, anterior division_R | T2a.R | 57.86 | -1.74 | -24.52 |
| 23 Middle Temporal Gyrus, posterior division_L | T2p.L | -60.95 | -27.39 | -10.91 |
| 24 Middle Temporal Gyrus, posterior division_R | T2p.R | 60.97 | -22.35 | -12.18 |
| 25 Middle Temporal Gyrus, temporooccipital part_L | TO2.L | -57.4 | -52.7 | 0.87 |
| 26 Middle Temporal Gyrus, temporooccipital part_R | TO2.R | 58.32 | -49.3 | 1.53 |
| 27 Inferior Temporal Gyrus, anterior division_L | T3a.L | -47.97 | -5.1 | -39.12 |
| 28 Inferior Temporal Gyrus, anterior division_R | T3a.R | 46.31 | -2.16 | -41.18 |
| 29 Inferior Temporal Gyrus, posterior division_L | T3p.L | -53.46 | -28.21 | -26 |
| 30 Inferior Temporal Gyrus, posterior division_R | T3p.R | 53.8 | -23.36 | -28.1 |
| 31 Inferior Temporal Gyrus, temporooccipital part_L | TO3.L | -51.81 | -53.45 | -16.68 |
| 32 Inferior Temporal Gyrus, temporooccipital part_R | TO3.R | 54.19 | -49.71 | -16.86 |
| 33 Postcentral Gyrus_L | POG.L | -38.53 | -27.78 | 51.5 |
| 34 Postcentral Gyrus_R | POG.R | 37.25 | -26.58 | 52.93 |
| 35 Superior Parietal Lobule_L | SPL.L | -29.28 | -49.4 | 57.63 |
| 36 Superior Parietal Lobule_R | SPL.R | 29.08 | -47.79 | 58.92 |
| 37 Supramarginal Gyrus, anterior division_L | SGa.L | -57 | -32.5 | 36.94 |
| 38 Supramarginal Gyrus, anterior division_R | SGa.R | 58.23 | -27.26 | 38.18 |
| 39 Supramarginal Gyrus, posterior division_L | SGp.L | -54.86 | -46.04 | 33.58 |
| 40 Supramarginal Gyrus, posterior division_R | SGp.R | 55.23 | -40.29 | 33.9 |
| 41 Angular Gyrus_L | AG.L | -50.45 | -55.74 | 29.3 |
| 42 Angular Gyrus_R | AG.R | 52.15 | -51.69 | 32.16 |
| 43 Lateral Occipital Cortex, superior division_L | OLs.L | -32.05 | -72.77 | 37.99 |
| 44 Lateral Occipital Cortex, superior division_R | OLs.R | 33.03 | -71.06 | 39 |
| 45 Lateral Occipital Cortex, inferior division_L | OLi.L | -45.21 | -75.63 | -1.94 |
| 46 Lateral Occipital Cortex, inferior division_R | OLi.R | 45.47 | -74.11 | -1.51 |
| 47 Intracalcarine Cortex_L | CALC.L | -10.47 | -74.92 | 8.19 |
| 48 Intracalcarine Cortex_R | CALC.R | 11.93 | -73.63 | 8.36 |
| 49 Frontal Medial Cortex_L | FMC.L | -5.38 | 43.84 | -17.87 |
| 50 Frontal Medial Cortex_R | FMC.R | 5.51 | 43.41 | -18.24 |
| 51 Supplementary Motor Cortex _L | SMC.L | -5.79 | -2.67 | 56.3 |
| 52 Supplementary Motor Cortex _R | SMC.R | 6.39 | -2.85 | 57.64 |
| 53 Subcallosal Cortex_L | SC.L | -5.7 | 20.6 | -15.68 |
| 54 Subcallosal Cortex_R | SC.R | 5.66 | 20.42 | -15.9 |
| 55 Paracingulate Gyrus_L | PAC.L | -6.82 | 36.58 | 20.93 |
| 56 Paracingulate Gyrus_R | PAC.R | 7.07 | 36.37 | 22.84 |
| 57 Cingulate Gyrus, anterior division_L | CGa.L | -5.15 | 18.19 | 24.6 |
| 58 Cingulate Gyrus, anterior division_R | CGa.R | 5.75 | 19.31 | 24.15 |
| 59 Cingulate Gyrus, posterior division_L | CGp.L | -6.3 | -38.56 | 28.79 |
| 60 Cingulate Gyrus, posterior division_R | CGp.R | 6.96 | -35.8 | 30.04 |
| 61 Precuneous Cortex_L | PCN.L | -8.16 | -60.06 | 37.25 |
| 62 Precuneous Cortex_R | PCN.R | 9.33 | -58.48 | 38.1 |
| 63 Cuneal Cortex_L | CN.L | -8.66 | -80.04 | 27.62 |
| 64 Cuneal Cortex_R | CN.R | 9.38 | -78.23 | 27.98 |
| 65 Frontal Orbital Cortex_L | FOC.L | -29.69 | 23.81 | -16.49 |
| 66 Frontal Orbital Cortex_R | FOC.R | 29.31 | 23.43 | -16.21 |
| 67 Parahippocampal Gyrus, anterior division_L | PHa.L | -21.68 | -9.28 | -30.7 |
| 68 Parahippocampal Gyrus, anterior division_R | PHa.R | 22.59 | -8.04 | -30.63 |
| 69 Parahippocampal Gyrus, posterior division_L | PHp.L | -22.15 | -32.31 | -17.13 |
| 70 Parahippocampal Gyrus, posterior division_R | PHp.R | 22.95 | -30.25 | -16.98 |
| 71 Lingual Gyrus_L | LG.L | -12.57 | -65.51 | -5.45 |
| 72 Lingual Gyrus_R | LG.R | 13.93 | -62.73 | -4.97 |
| 73 Temporal Fusiform Cortex, anterior division_L | TFa.L | -32.3 | -4.53 | -41.6 |
| 74 Temporal Fusiform Cortex, anterior division_R | TFa.R | 30.87 | -2.55 | -42.28 |
| 75 Temporal Fusiform Cortex, posterior division_L | TFp.L | -36.02 | -29.45 | -25.04 |
| 76 Temporal Fusiform Cortex, posterior division_R | TFp.R | 36.53 | -23.81 | -28.05 |
| 77 Temporal Occipital Fusiform Cortex_L | TOF.L | -33.32 | -53.65 | -15.95 |
| 78 Temporal Occipital Fusiform Cortex_R | TOF.R | 35.02 | -49.88 | -16.56 |
| 79 Occipital Fusiform Gyrus_L | OF.L | -26.33 | -76.86 | -13.45 |
| 80 Occipital Fusiform Gyrus_R | OF.R | 27.24 | -75.48 | -12.29 |
| 81 Frontal Operculum Cortex_L | FO.L | -39.82 | 18.29 | 4.62 |
| 82 Frontal Operculum Cortex_R | FO.R | 41.14 | 18.82 | 4.75 |
| 83 Central Opercular Cortex_L | CO.L | -48.03 | -8.28 | 11.64 |
| 84 Central Opercular Cortex_R | CO.R | 49.47 | -5.66 | 11.1 |
| 85 Parietal Operculum Cortex_L | PO.L | -48.4 | -31.53 | 20.3 |
| 86 Parietal Operculum Cortex_R | PO.R | 48.86 | -27.69 | 21.65 |
| 87 Planum Polare_L | PP.L | -46.77 | -5.34 | -7.54 |
| 88 Planum Polare_R | PP.R | 48.11 | -3.56 | -7.1 |
| 89 Heschl's Gyrus_L (includes H1 and H2) | H.L | -45.22 | -20.04 | 7.31 |
| 90 Heschl's Gyrus_R (includes H1 and H2) | H.R | 46.04 | -17.36 | 6.89 |
| 91 Planum Temporale_L | PT.L | -52.64 | -29.69 | 10.8 |
| 92 Planum Temporale_R | PT.R | 54.84 | -25.33 | 12.39 |
| 93 Supracalcarine Cortex_L | SCLC.L | -12.29 | -68.86 | 15.57 |
| 94 Supracalcarine Cortex_R | SCLC.R | 9.09 | -74.03 | 14.47 |
| 95 Occipital Pole_L | OP.L | -17.23 | -96.36 | 7.23 |
| 96 Occipital Pole_R | OP.R | 18.09 | -95.14 | 8.35 |
| 97 Thalamus_L | Thal.L | -9.99 | -19.16 | 6.28 |
| 98 Thalamus_R | Thal.R | 10.92 | -18.5 | 6.6 |
| 99 Caudate_L | Caud.L | -12.84 | 8.92 | 9.71 |
| 100 Caudate_R | Caud.R | 13.5 | 9.66 | 10.87 |
| 101 Putamen_L | Put.L | -24.79 | 0.54 | 0.42 |
| 102 Putamen_R | Put.R | 25.48 | 2.03 | 0.35 |
| 103 Pallidum_L | Pall.L | -19.13 | -5.17 | -1.47 |
| 104 Pallidum_R | Pall.R | 19.75 | -3.87 | -1.44 |
| 105 Hippocampus_L | Hip.L | -25.18 | -23.25 | -13.92 |
| 106 Hippocampus_R | Hip.R | 26.5 | -20.99 | -14.07 |
| 107 Amygdala_L | Amy.L | -22.86 | -5.18 | -17.49 |
| 108 Amygdala_R | Amy.R | 22.77 | -3.69 | -17.91 |
| 109 Accumbens_L | Accbns.L | -9.34 | 11.14 | -7.11 |
| 110 Accumbens_R | Accbns.R | 9.21 | 11.46 | -6.43 |
