# Supplementary material for: Disrupted White Matter Integrity and Structural Brain Networks in Temporal Lobe Epilepsy With and Without Interictal Psychosis
Source: Front Neurol. 2020 Sep 24;11:556569. doi: 10.3389/fneur.2020.556569 (PMC7542674; doi:10.3389/fneur.2020.556569)
Supplement: Supplementary file 3 [file Table_3.DOCX]

Supplementary Table 3. The list of involved nodes in the impaired local networks in Fig. 3 and 4.

| **AAL** | |  | **HOA** | |
| --- | --- | --- | --- | --- |
| ***TLE-P < HC*** | |  | ***TLE-P < HC*** | |
| p<0.001 | PreCG.L, PreCG.R, SFGdor.R, ORBsup.L, ORBsup.R, MFG.L, MFG.R, ORBmid.R, IFGtriang.L, IFGtriang.R, ORBinf.R, ROL.R, SMA.L, SMA.R, SFGmed.L, SFGmed.R, ORBsupmed.L, REC.L, REC.R, INS.R, ACG.L, DCG.L, DCG.R, PCG.L, HIP.L, HIP.R, PHG.L, PHG.R, AMYG.L, AMYG.R, CAL.L, CAL.R, CUN.L, CUN.R, LING.L, LING.R, SOG.R, MOG.L, MOG.R, IOG.R, FFG.L, FFG.R, PoCG.R, IPL.L, IPL.R, ANG.R, PCUN.L, PCUN.R, PCL.L, PCL.R, CAU.L, CAU.R, PUT.R, THA.L, THA.R, STG.R, TPOsup.L, TPOsup.R, MTG.R, TPOmid.L, ITG.R |  | p<0.001 | FP.L, FP.R, F1.L, F2.L, F3t.L, F3t.R, F3o.L, F3o.R, TP.L, TP.R, T2p.L, T3a.L, T3p.L, TO3.L, OLs.L, OLi.L, FMC.L, FMC.R, SMC.L, SMC.R, SC.R, PAC.L, PAC.R, CGa.L, CGa.R, CGp.L, CGp.R, PCN.L, PCN.R, FOC.L, FOC.R, PHa.L, LG.L, LG.R, TFp.L, TOF.L, OF.L, FO.L, PO.L, PP.L, H.R, PT.L, SCLC.L, SCLC.R, OP.L, Thal.L, Thal.R, Caud.R, Put.L, Put.R, Pall.R, Hip.L, Amy.L, Accbns.L, Accbns.R |
|  |  |  | p=0.012 | T2p.R, TO3.R, SGp.R, AG.R, OLs.R, OLi.R, PT.R |
| ***TLE-NonP < HC*** | |  | ***TLE-NonP < HC*** | |
| p=0.004 | SFGmed.R, ORBsupmed.L, ACG.L, ACG.R, DCG.L, DCG.R, PCG.L, CAL.L, CUN.L, PCUN.L, PCUN.R |  | p<0.001 | T3a.L, PAC.L, CGa.L, CGp.L, PCN.L, PCN.R, CN.R, PHa.L, PHp.L, PHp.R, LG.L, TFa.L, TFp.L, TFp.R, SCLC.L, SCLC.R, Thal.L, Hip.L, Hip.R, Amy.L, Accbns.L |
| p=0.010 | HIP.L, PHG.L, AMYG.L, LING.L, FFG.L, THA.L, MTG.L, TPOmid.L |  |  |  |
| ***TLE-P < TLE-NonP*** | |  | ***TLE-P < TLE-NonP*** | |
| p=0.029 | ORBsup.L, REC.L, HIP.L. LING.L, SPG.L, THA.L, TPOsup.L |  | p=0.019 | FP.L, TP.L, TOF.L, PP.L, Put.L, Hip.L, Accbns.L |

* The node abbreviations correspond to those of Supplementary Table S1 and S2.
